# The effects of psychological distress after surgery in patients undergoing lumbar spinal fusion

**DOI:** 10.1186/s12891-024-07364-7

**Published:** 2024-04-13

**Authors:** Qiang Li, Jian Luan, Yong Lin, Meng Kong, Xinhu Guo, Jindong Zhao

**Affiliations:** 1 Department of Spine Surgery, Qingdao Municipal Hospital Group, No.5 Donghai Middle Road, Shinan District, Qingdao, 266000 China; 2 Department of Orthopaedics, Peking University Third Hospital, No.49 North Garden Road, Haidian District, Beijing, 100191 China

**Keywords:** Psychological distress, MSPQ, Lumbar spinal fusion, ODI, JOA, VAS

## Abstract

**Background:**

The aim of this study was to evaluate the psychological distress pre-operatively, at 3, 6, and 12 months in patients who underwent lumbar spine fusion surgery.

**Methods:**

A total of 440 patients received instrumented lumbar spine fusion were enrolled. Psychological distress was evaluated using the Modified Somatic Perception Questionnaire (MSPQ) and the Modified Zung Depressive Index (ZDI). The results of lumbar fusion surgery were evaluated using the Oswestry Disability Index (ODI), the Japanese Orthopedic Association (JOA-29), and the visual analog scale (VAS).

**Results:**

Psychological distress was reported among 23% of patients and 7, 5.5, and 4.0% of the patients preoperatively, at 3, 6, and 12 months after lumbar surgery, respectively. The mean MSPQ score decreased from 8.78 (before surgery) to 4.30, 3.52, and 3.43 at 3, 6 and 12 months in after surgery, respectively, in patients with psychological distress patients (*p* < 0.001). The mean ZDI score decreased from 17.78 to 12.48, 10.35, and 9.61 (*p* < 0.001). The mean ODI score decreased from 22.91 to 11.78, 10.13, and 9.96 (*P* < 0.001). The mean JOA score increased from 13.65 to 22.30, 23.43, and 23.61 (*P* < 0.001). The mean low back pain (LBP) VAS score decreased from 4.48 to 1.96, 1.52, and 1.51 (*P* < 0.001); moreover, the mean leg pain (LP) VAS score decreased from 5.30 to 1.30, 1.04, and 1.03 (*P* < 0.001).

**Conclusions:**

Patients with psychological distress may experience surgical intervention benefits equal to those of ordinary patients. Moreover, reduced pain and disability after surgical intervention may also alleviate psychological distress. Hence, we highly recommend that patients with psychological distress undergo surgical intervention as normal patients do, but appropriate screening measures and interventions are necessary.

## Introduction

Psychological distress is reported as a common condition for people with constant trouble in the back and leg after lumbar fusion. Low back pain (LBP) and leg pain (LP) continue to be one of the top 3 causes of chronic pain in the United States [1–3]. It is reported that almost everyone suffers from LBP in their lifetime, and approximately 4–33% of worldwide population is suffering at any given time. Currently in China, Jin et al. reported that the 1-year prevalence rate of LBP among workers and teachers was 50%, while among the rural population the rate increased to 64% [2, 3]. It greatly influences the quality of life, and great effort has been made in recent years to develop safe and effective treatment options for LBP and LP. Recent advances in the development of new surgical techniques and technologies to treat LBP have significantly increased rates of lumbar spine surgery over the past 25 years.

Psychological distress can affect spine surgery outcomes, and patients with chronic LBP, often have an abnormal psychological profile [4, 5]. A previous study indicated a stepwise increase in psychological distress from the simplest state in the majority of people without backaches to the greatest in those who have chronic LBP and numbness pain in the lower limbs [6]. Despite the evident correlation observed between chronic LBP and psychological distress, the exact rationale underlying this association is not clear. According to certain researchers, mental discomfort can be a risk factor for backache, but people else come upon the opinion that mental wellness constitutes an outcome of LBP [7].

Lumbar spine fusion surgery can be recommended for several disorders causing chronic LBP and LP if patients complain of severe symptoms despite conservative treatment. Nevertheless, lumbar vertebral fusion procedure may be advised. For several symptomatic illnesses, such as spinal stenosis, surgical intervention is associated with successful outcomes and is affordable [8]. In contrast, individuals experiencing persistent discogenic LBP, which is a complex and multi-factorial clinical condition, need thorough radiological investigation before surgical interventions are recommended. It was previously indicated that systematic psychological treatment might produce more positive results than fusion procedures under such clinical conditions [9].

It has been reported that depression, anxiety, thought patterns and personality styles can predict the outcome of spinal surgery [6, 10–12]. In contrast to those without mental health issues, individuals with depression who had received surgery to decompress over spinal obstruction experienced greater discomfort, lower levels of medical fulfillment, less independence, and poorer levels of enjoyment of life following operation [13]. However, there are few prior researches regarding the consequences of spinal fusion procedures evaluated by Modified Somatic Perception Questionnaire (MSPQ) and Modified Zung Depressive Index (ZDI).

The aim of this prospective study was to evaluate psychological distress in patients before surgery, and at 3, 6, and 12 months after surgery and to investigate relationship between distress and functional disability.

## Methods

### Subjects

The present study utilized vertebral records collected at Peking University Third Hospital and Qingdao Municipal Hospital. Preoperative and surgical monitoring information at 3, 6, and 12 months was gathered systematically as a component of doctors’ ordinary surgical routine. A total of 440 successive recipients who received non-urgent configured spinal union were enrolled. The cohort identified for this study included patients who underwent posterolateral lumbar fusion (PLF), posterior lumbar interbody fusion (PLIF), or transforaminal interbody fusion (TLIF). A total of 250 females (56.8%) and 190 males (43.2%) with a mean age of 59.76 ± 13.50 years were included in the cohort. Patients with broken bones, tumors, tuberculosis, neurological or congenital spinal curvature, and revision surgery were excluded. Among them, 404 patients provided complete follow-up information from all the examinations.

Radiologically (X-ray, MRI, or CT) identified neurological disease and grave, long-term discomfort in the back and the radicular leg, meeting the requirements for operation. The patients with vertebral curvature, lumbar disc herniation, degenerative spondylolisthesis, and progressive deformity constituted the primary reasons for fusion surgery in 316 (78.2%), 44 (10.9%), 22 (5.45%), and 22 (5.45%) people, respectively. Vertebral fusion was accomplished with PLF, PLIF and TLIF. All patients had a history of more than six months of non-effective response to conservative treatment (such as physical therapy, medication, and epidural injection).

### Psychological distress

The MSPQ and the Modified ZDI are utilized in the standardized emotional Distress and Risk Assessment Method (DRAM), which was developed by Main et al. [14]. The DRAM was created to provide an immediate method to check for mental health issues and to notify medical clinicians of possible requirements for further, thorough mental health evaluations. Four qualitative sections are provided by the survey and depend on the emotional categorization of the individuals: Type N (normal; lack of anxiety or unusual illness-related activity); Type R (at danger; better results, mostly in mood disorders complaints); Type DD (distressed-depressive; better results across the board, with particularly excellent for anxious symptomatology); and Type DS (distressed-somatic; excellent results across the board, specifically for sensory sensibility). For the most extensive emotional examination, the DRAM was successfully established and found to correspond with deteriorating mental illness [15]. Additionally, adverse results in the management of backache are linked to DRAM scores showing greater emotional disturbance [14].

### Conditions, distress, and related elements

Disability was measured pre- and post-operatively using the Oswestry Disability Index (ODI). The Japanese Orthopedic Association (JOA-29) score was used to assess the quality of life of patients with the lumbar degenerative disease before lumbar fusion and post-operation, the back and leg pain was assessed with the Visual analog scale (VAS).

The ODIone of the most widely used gold standards of outcome measurement in patients with LBP, has been used for more than 25 years and is currently translated into at least 15 different languages [16–18]. Compared with other advantages, its ease of use, high reliability, and validity make it suitable for most patients in mainland China [19]. The ODI score was used to assess the quality of life of patients with degenerative diseases before lumbar fusion and after surgery. The ODI comprises 10 items that assess the level of pain interference with physical activity. Each item is scored from 0 to 5; 5 represents the greatest disability. The minimum value of the ODI score was considered negative below 6 and the maximum value was 50. Higher ODI score indicates a worse outcome.

The JOA score was developed by JOA members in 1986 to assess LBP [20]. The simplified Chinese version JOA total score is 29, with: 9 points for subjective symptoms, 6 points for clinical symptoms, and 14 points for daily activity limitation. Bladder function (− 6 to 0): 0, normal; −3, mild limitation; −6, urinary incontinence or urinary retention. The highest potential score is 29 points: <10, poor; 10–15, moderate; 16–24, good; 25–29, excellent. The improvement index reflects the improvement in the lumbar function of patients before and after treatment [21]. The JOA-29 is reliable and valid for use with LBP patients.

LBP and LP were assessed with a segmented numeric version of the VAS, on which a respondent selects a number (from 0 to 10) that best reflects the intensity of their pain. Respondents are asked to report their average pain intensity for the last week; 0 represents no pain and 10 represents the worst pain imaginable the visual analog scale [22].

A survey additionally collected biographical information about the individuals receiving treatment, such as age, sex, years of education, employment status (working, unemployed, retired, or on sick leave), smoking, duration of symptoms, and history of previous lumbar spine surgery.

## Results

There were 404 participants who completed the DRAM survey (Table 1). Pre-operatively 23% of patients, whose ZDI scores were > 17 points and/or MSPQ scores > 12 was classified as having psychological distress (Table 2) [14]. The corresponding data 3, 6, and 12 months after surgery were 7%, 5.5%, and 4.0%, respectively (two patients had wound infections, three patients still had pre-operative clinical symptoms and the other three patients had psychological problems). Before surgery, psychological distress was significantly higher in females (31.3%) than in males (11.5%) (*P* < 0.01); however, at 12 months after surgery, the difference between females (3.7%), and males (3.4%) disappeared (*P* > 0.05). In addition, there was no significant difference in age or educational qualification between patients with and without psychological distress.

**Table 1:** Descriptive features of demographics (*n* = 404)

Normal patients	59.84 ± 11.18
Psychological distress (Years, SD)	62.40 ± 9.60
Sex (n, %)	
Female	230 (56.9%)
Male	174 (43.1%)
Education years mean (SD	
Normal patients	10.63 ± 4.12
Psychological distress	9.20 ± 3.77
At working	58 (14.4%)
Retired	218 (54%)
Sick leave	12 (3%)
Unemployed	64 (15.8%)
Housewives	52 (12.9%)
Duration of symptoms, months (n, %) Mean (SD)	
Leg pain	44(10.9%)41.47 ± 106.98
Low back pain and leg pain	360(89.1%) 57.12 ± 73.08
Indication for fusion (n, %)	
Spinal stenosis	316 (78.2%)
Disc herniation or degeneration	44 (10.9%)
Degenerative spondylolisthesis	22 (5.45%)
Degenerative scoliosis	22 (5.45%)

*Note* Summary statistics are given as mean (SD) or percentages

**Table 2:** Results of distress and risk assessment method (DARM) questionnaire

Category	*N*=404Pre-	Post-3 months	Post-6 months	post-12 months
Normal	312	376	382	388
R	60 (F,44; M,16)	20(F,12; M,8)	16(F,10; M,6)	12(F,6; M,6)
DD	20(F,20)	6	4	2
DS	12(F,8;M,4)	2(F)	2(F)	2(F)

*Note* F (female), M (male)

Type N: modified Zung< 17

Type R: modified Zung 17-33 and MSPQ < 12

Type DD: modified Zung> 33

Type DS: modified Zung 17-33 and MSPQ > 12

**Fig. 1:**
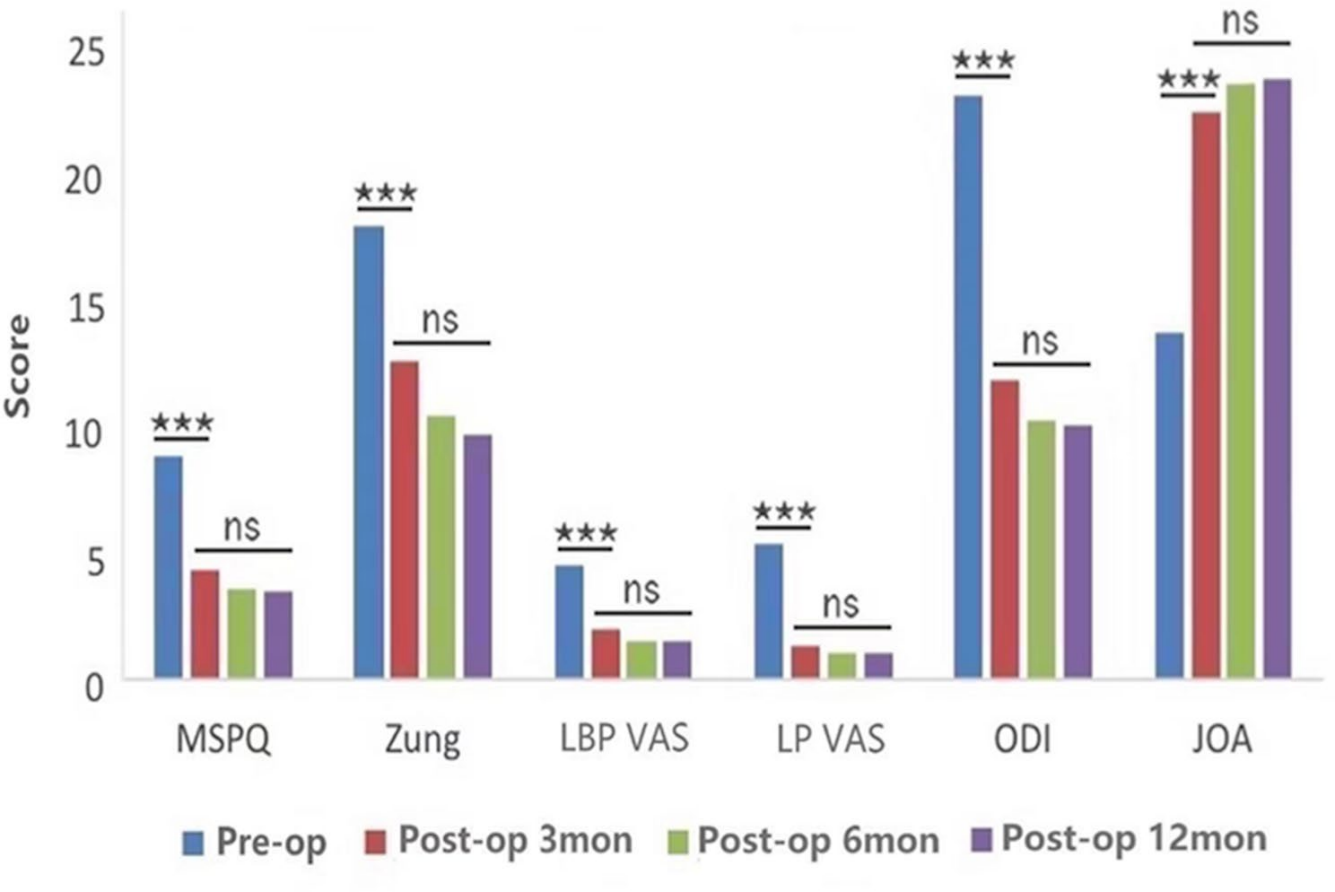
The MSPQ, Zung, LBP, LP, ODI, and JOA scores were significantly different among patients who had psychological distress pre-operatively, and at 3, 6, and 12 months (*P* < 0.001), moreover, there was no significant difference between the groups at any of time points after lumbar surgery. (*** means *P* < 0.001, ns means *P* > 0.05)

**Fig. 2:**
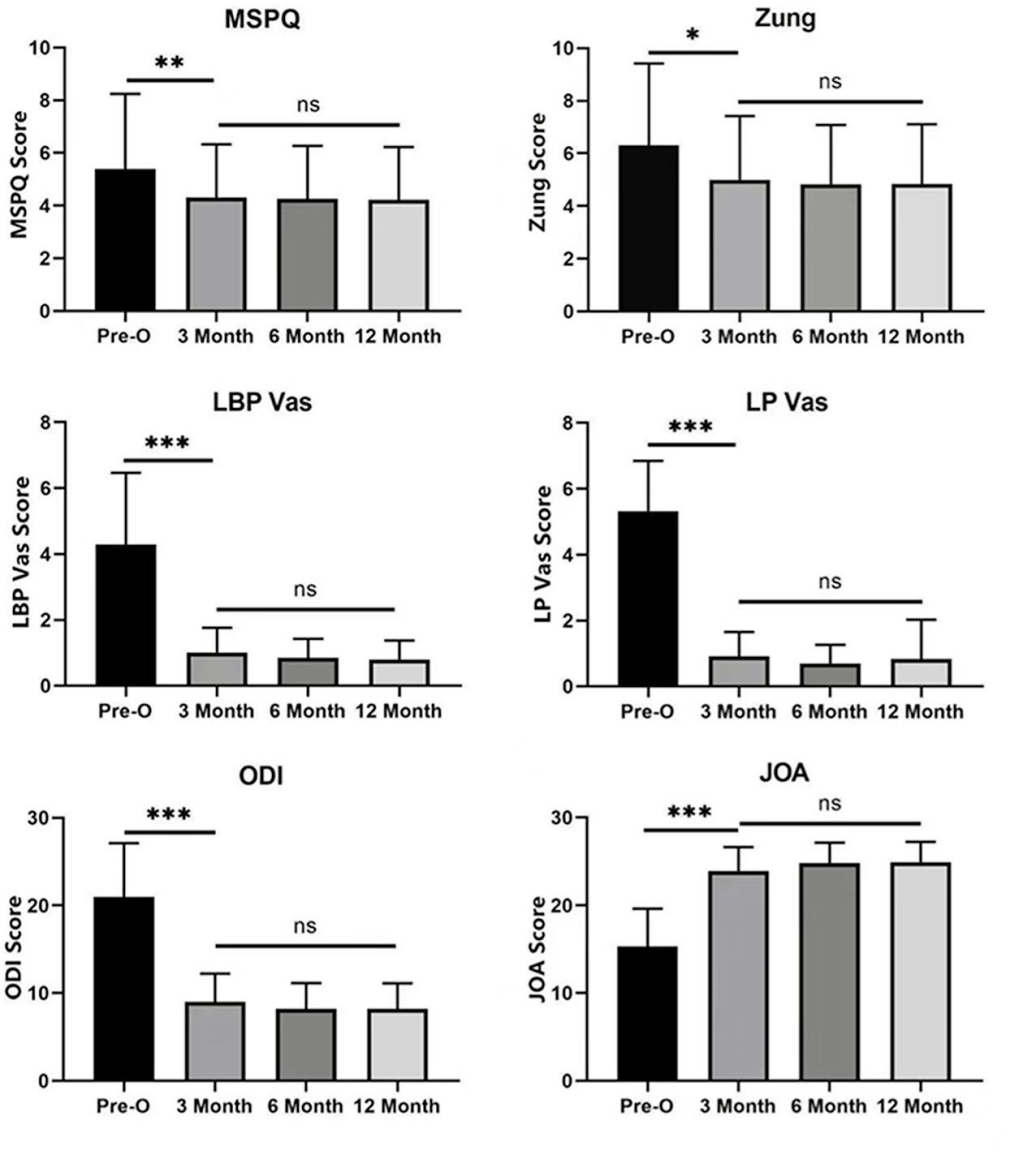
LBP, LP, ODI, and JOA scores were significantly different among patients without psychological distress pre-operatively, and at 3, 6, and 12 months (*P* < 0.001), as were MSPQ scores (*P* < 0.01), and Zung scores (*P* < 0.05). Similarly, there was no significant difference between the groups at different time points after lumbar surgery. (*means *P* < 0.05, **means *P* < 0.01 and ***P < 0.001)

In patients with or without psychological distress, there were significant differences in VAS, ODI, and JOA-29 scores before surgery and at 3, 6, and 12 months after surgery (*P* < 0.05 Figs. 1 and 2). However, there was no significant difference between the groups at different time points after lumbar surgery (*P* > 0.05).

**Fig. 3:**
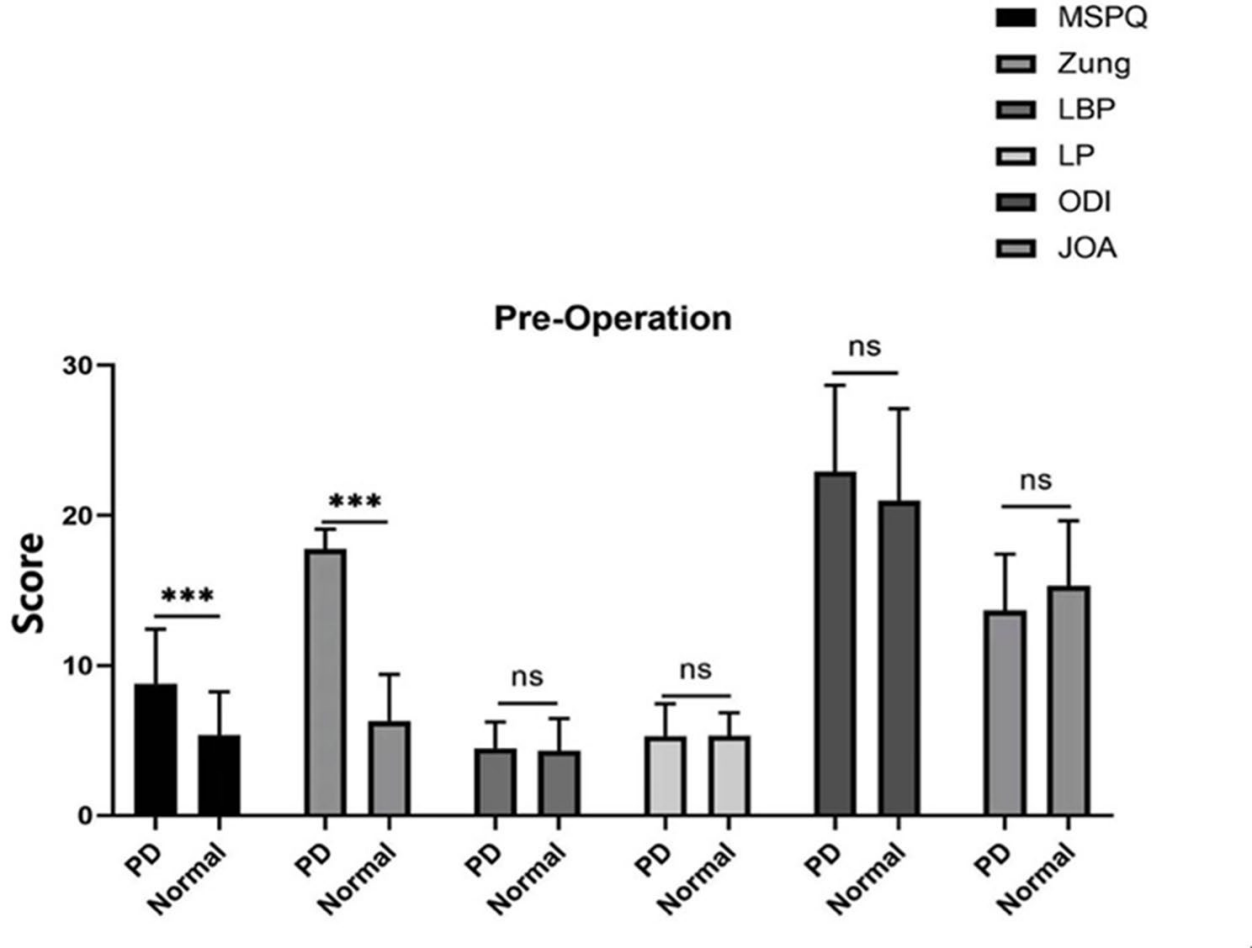
The MSPQ and Zung scores were significantly different (*P* < 0.001) before the operation. (PD means patients with psychological distress, and Normal means patients without psychological distress)

**Fig. 4:**
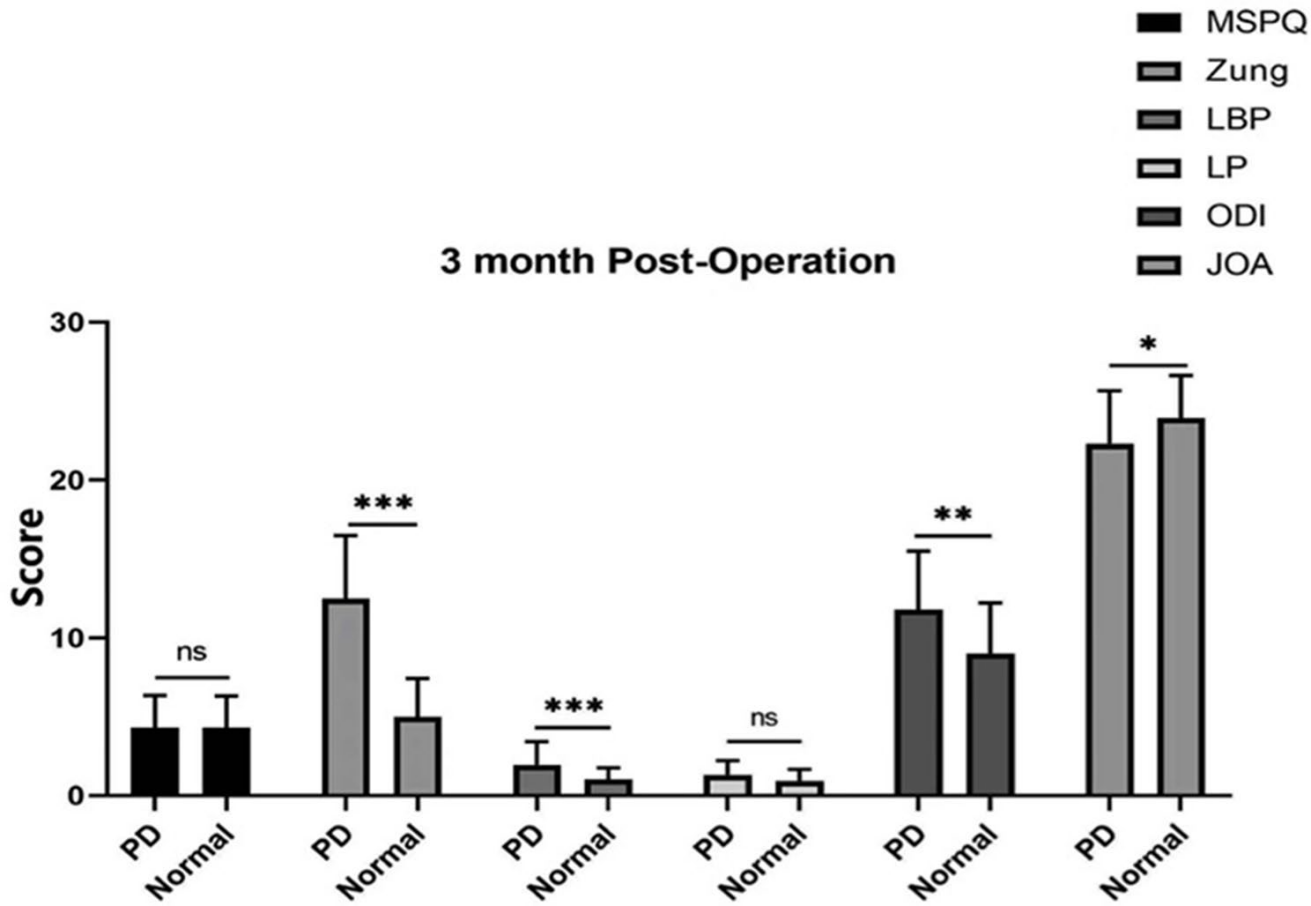
Zung, LBP, ODI, and JOA scores were significantly different between PD patients and normal controls at 3 months (*P* < 0.05)

**Fig. 5:**
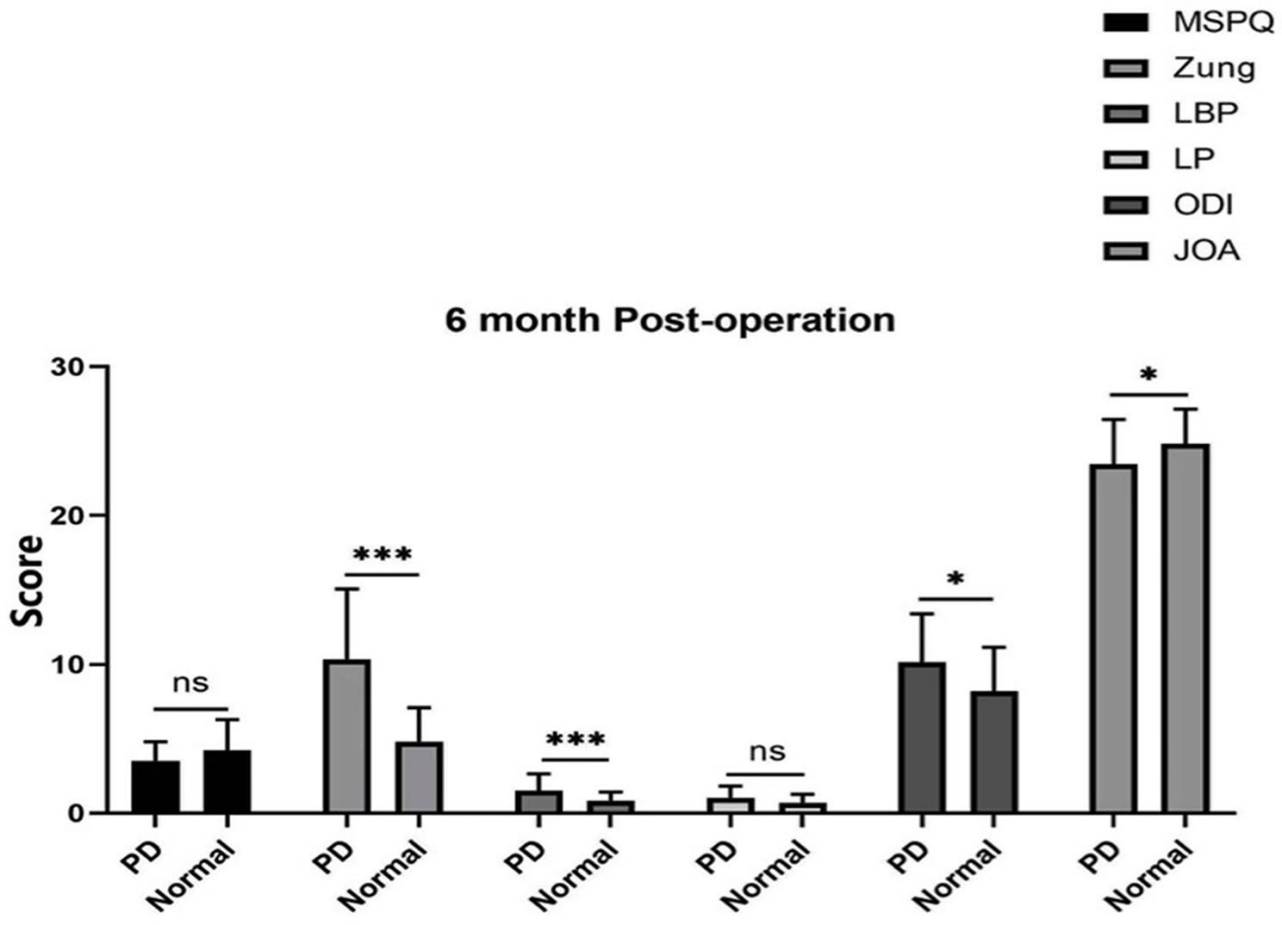
Zung and LBP scores were significantly different between PD patients and normal controls at 6 months

**Fig. 6:**
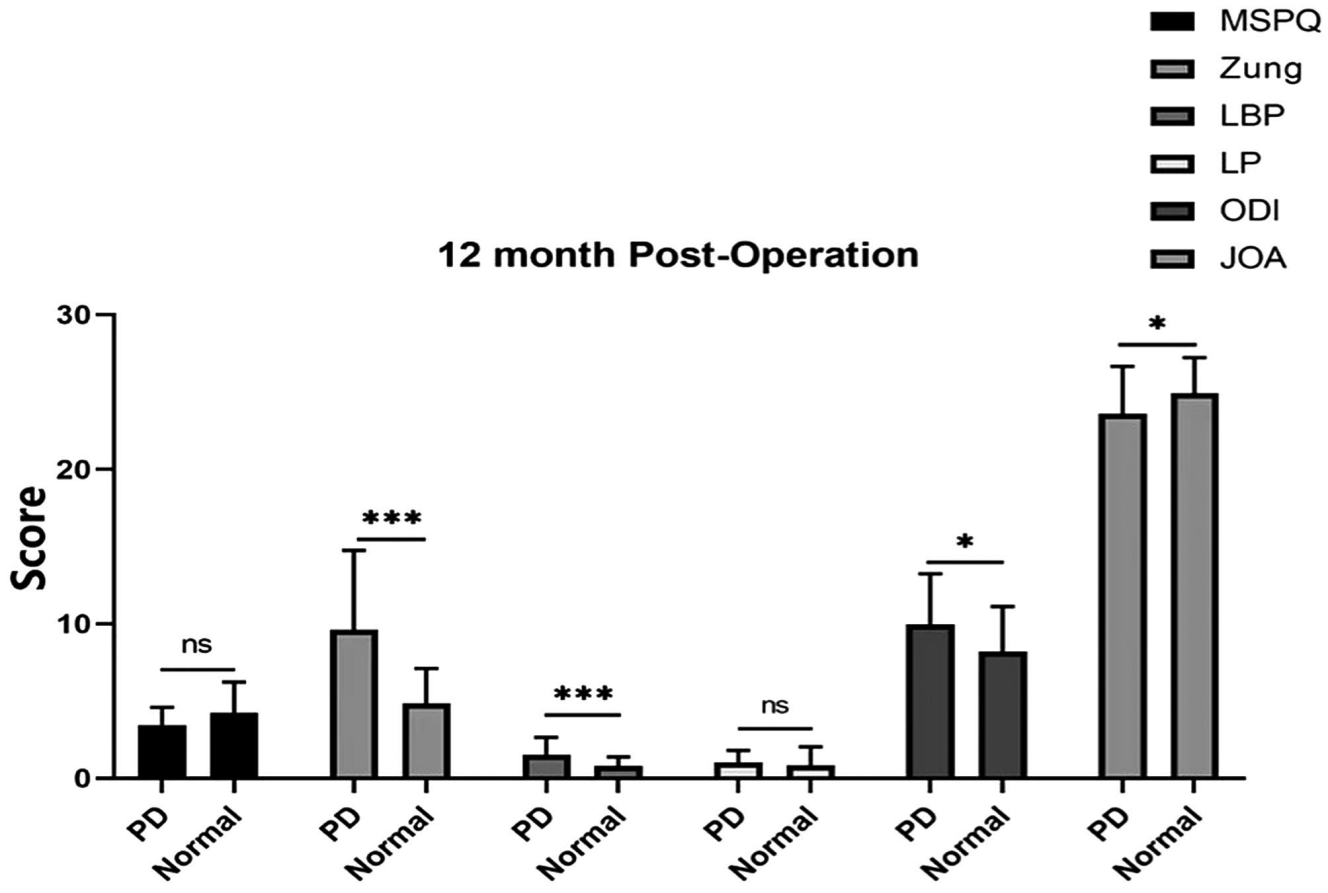
Zung, LBP, ODI, and JOA scores were significantly different between PD patients and normal controls at 12 months

The MSPQ and Zung scores were significantly different before the operation between patients with psychological distress and those in the normal group (Fig. 3). Moreover, the Zung and LBP scores were significantly different between the psychological distress group and the normal group at 3, 6, and 12 months (*P* < 0.001), ODI score was significantly different at 6 months (*P* < 0.01), ODI and JOA scores were significantly different at 6 and 12 months (*P* < 0.05), whereas the MSPQ and LP scores were not significantly difference at 3, 6, and 12 months (*P* > 0.05 Figs. 4, 5 and 6).

## Discussion

The biopsychosocial framework for healthcare acknowledges the intricate relationships between a patient’s physiological state or sickness, mental state and interpretation of the illness, and any relevant contextual variables [23, 24]. The mental state of an individual may impact their physiological well-being and inversely; the state of their body may impact their life irrespective of the physiological illness procedure. These ideas are vital since mental health problems can influence how well a treatment works [25]. Multiple investigations [12, 14, 23] indicate that interpersonal variables exert a detrimental impact on the treatment of vertebral illnesses. The DRAM survey confirmed the adverse effects of anxiety on therapeutic results, and has been demonstrated in prior research published [14, 26]. However, numerous lumbar doctors remain unaware of these feasible repercussions.

In the present study, psychological distress and the factors associated with them were evaluated from the register-based data on patients undergoing lumbar spine fusion. This study was to evaluate the psychological distress of the patients and to determine exactly to what extent this distress affects the outcomes of lumbar spinal fusion. The results of this study showed that mental disorders were more common in people with long-term LBP and LP but improved at 12months after the operation. The scores for body disorders (LBP, ODI, and JOA) improved after the operation, but were still slightly poorer in the psychological distress group than in the normal group at 3, 6, and 12-month follow-ups. It seems that psychological distress interferes with a few adverse surgical results for patients after lumbar spinal fusion. These data indicate that psychological distress has some effect on the outcomes of lumbar spinal fusion. However, in the psychological distress group, the VAS, ODI, and JOA score were significantly improved after surgery than they were before surgery. These findings showed that patients’ disability has greatly improved even in the psychological distress group.

Our discoveries agree with the findings from various reported researches [14, 27]. In our research, 23% of the patients had some psychological distress, like the 28% found by Grevitt et al. [27], the 29% found by Main et al. [14], and the 23% found by Greenough and Fraser [28]. This indicates that nearly a quarter of people with chronic low back pain suffering from a psychological distress, which is a social problem that cannot be ignored. Therefore, we believe that relieving low back pain in patients may reduce their psychological distress.

The VAS, ODI, and JOA-29 scores were significantly different before and after surgery at 3, 6, and 12 months of follow-up. These results indicate that disability significantly improved in patients with or without psychological distress after surgery. These changes suggested that chronic LBP and LP were alleviated through lumbar fusion surgery. Additionally, pre-operative fear and unknown surgical outcomes can also lead to psychological distress. However, 4.0% of patients still experienced psychological distress, probably due to poor surgical outcomes (such as wound infections and unrelieved symptoms) and due to the patient’s own family circumstances (for example, the son of one male patient got depression and dropped out of college). In general, the operation significantly relieved the patient’s long-term LBP and LP and improved the associated psychological distress. Consequently, patient’s quality of life has significantly improved enabling them to return to work early after post-operative recovery and reducing economic and social burdens.

In addition, chronic pain and disability may cause psychological distress. Mental health issues, such as symptoms of sadness, were shown to be a marker of lower operational skills following vertebral fusion operation in the study by Trief et al. [12]. At 3-, 6-, and 12-month monitoring in the current inquiry, individual alterations in ODI scores were considerably reduced. Our results disagree with the findings presented by Trief et al. In the present study, participants regardless of their mental health status, experienced a considerable reduction in limitations after the procedure, and this decline had a strong correlation with a decline in anxiety and depression. The aforementioned findings support our own earlier claims that emotional suffering might get alleviated with operation in addition to bodily discomfort such as suffering or incapacity.

Chronic LBP and LP are one of the most grievous disease conditions causing sick leaves and early retirements in Western countries [29]. In the present study, most of the patients were retired at baseline. However, although the age of the patients was similar in both groups, significantly fewer depressive patients were still at work than other patients. Furthermore, we observed that patients with psychological depressive symptoms were less educated than normal patients. Naturally, people with less education may be doing heavier work and hence earlier retirement from their jobs. However, this may also reflect the psychosocial factors attached to the psychological distress symptoms.

This research has certain limitations. A 45-item patient questionnaire serves as the foundation for a diagnosis of psychological distress, and is administered mainly through the spinal specialist clinic and telephone conversations. These procedures were inevitable to avoid some misunderstandings during information-gathering and collection of ambiguous data. Even through the spinal specialist clinic and telephone conversations, some data will be omitted, but this does not prevent DRAM from being a dependable screening tool for psychological distress. Furthermore, the 12-month monitoring period is inadequate given that spinal union consequences have been discovered to persist for as long as five decades [30]. The study’s variables alone are unable to account for the alterations in the participants’ emotions, and further longitudinal studies are probably needed to determine the connections between mental issues and impairment in the vertebral fusion procedure. It seems logical to evaluate the groups independently in the current investigation because the number of subgroups was still quite small.

## Conclusions

Patients who undergo vertebral fusion operation for persistent LBP and LP frequently experience psychological discomfort. Surgical interventions in people with psychological distress were successful, similar to those in other patients, and had effective outcomes with at least 12 months of follow-up. A reduction in discomfort and limitations following operation can additionally decrease emotional suffering. Overall, we highly recommend that patients with psychological distress undergo surgical intervention and suggest appropriate screening measures and interventions throughout the lead-up period to and after the operation.

## Data Availability

The data and material that support the findings of this study are available upon request from the corresponding author upon reasonable request.

## Abbreviations

MSPQ: Modified Somatic Perception Questionnaire
ZDI: Modified Zung Depressive Index
ODI: Oswestry Disability Index
JOA-29: Japanese Orthopedic Association
VAS: Visual analog scale
LBP: Low back pain
LP: Leg pain
PLF: Posterolateral lumbar fusion
PLIF: Posterior lumbar interbody fusion
TLIF: Transforaminal interbody fusion
DRAM: Distress and Risk Assessment Method
PD: means patients with psychological distress
Normal: means patients without psychological distress
Type N: normal
Type R: at danger
Type DD: distressed-depressive
Type DS: distressed-somatic
